# Estimating Departure Time Using Thermal Camera and Heat Traces Tracking Technique

**DOI:** 10.3390/s20030782

**Published:** 2020-01-31

**Authors:** Ziyi Xu, Quchao Wang, Duo Li, Menghan Hu, Nan Yao, Guangtao Zhai

**Affiliations:** 1 Shanghai Key Laboratory of Multidimensional Information Processing, East China Normal University, Shanghai 200241, China; 10185000321@stu.ecnu.edu.cn (Z.X.); 10181511228@stu.ecnu.edu.cn (Q.W.); 2 School of Statistics, East China Normal University, Shanghai 200241, China; 3 School of Mathematical Sciences, East China Normal University, Shanghai 200241, China; 4 Hangzhou HIKVISION Digital Technology Co., LTO., Hangzhou 310051, China; liduoee@gmail.com; 5 Institute of Image Communication and Information Processing, Shanghai Jiao Tong University, Shanghai 200240, China; zhaiguangtao@sjtu.edu.cn; 6 Key Laboratory of Artificial Intelligence, Ministry of Education, Shanghai 200240, China; 7 Shanghai Jianglai Data Technology Co., Ltd, Shanghai 200241, China; yaon@jianglaidata.com

**Keywords:** thermal imaging, criminal investigation, Newton’s Law of Cooling, heat traces analysis

## Abstract

Advancement in science and technology is playing an increasingly important role in solving difficult cases at present. Thermal cameras can help the police crack difficult cases by capturing the heat trace on the ground left by perpetrators, which cannot be spotted by the naked eye. Therefore, the purpose of this study is to establish a thermalfoot model using thermal imaging system to estimate the departure time. To this end, in the current work, we use a thermal camera to acquire the thermal sequence left on the floor, and convert it into the heat signal via image processing algorithm. We establish the model of thermalfoot print as we observe that the residual temperature would exponentially decrease with the departure time according to Newton’s Law of Cooling. The correlation coefficients of 107 thermalfoot models derived from the corresponding 107 heat signals are basically above 0.99. In a validation experiment, a residual analysis is conducted and the residuals between estimated departure time points and ground-truth times are almost within a certain range from −150 s to +150 s. The reverse accuracy of the thermalfoot model for estimating departure time at one-third, one-half, two-thirds, three-fourths, four-fifths, and five-sixths capture time points are 71.96%, 50.47%, 42.06%, 31.78%, 21.70%, and 11.21%, respectively. The results of comparison experiments with two subjective evaluation methods (subjective 1: we directly estimate the departure time according to obtained local curves; subjective 2: we utilize auxiliary means such as a ruler to estimate the departure time based on obtained local curves) further demonstrate the effectiveness of thermalfoot model for detecting the departure time inversely. Experimental results also demonstrated that the thermalfoot model has good performance on the departure time reversal within a short time window someone leaves, whereas it is probably only approximately 15% to accurately determine the departure time via thermalfoot model within a long time window someone leaves. The influence of outliers, ROI (Region of Interest) selection, ROI size, different capture time points and environment temperature on the performance of thermalfoot model on departure time reversal can be explored in the future work. Overall, the thermalfoot model can help the police solve crimes to some extent, which in turn brings more guarantees for people’s health, social security, and stability.

## 1. Introduction

The social change brought by the development of science and technology is two-sided. With the development of science and technology, criminal means are becoming more and more diversified, criminals’ anti-reconnaissance consciousness is becoming stronger and stronger, and crime scenes are becoming more and more complicated. Some difficult cases in the past remain unsolved due to the limitations of technology at that time. In the trend of high-tech crime, the police’s criminal investigation ability is facing new breakthrough challenges. In the context of the intelligent era, to meet people’s demand for the secure and stable development of society, many scientists in related fields have studied several advanced criminal investigation means, such as forensic DNA analysis technology [1,2], polygraph detection technology based on information analysis [3,4], and trace material evidence identification technology [5,6]. DNA analysis technology utilizes the DNA left at crime scenes to identify criminals with high matching degree and sensitivity. However, it is likely for some extreme situations that the amount of DNA at the crime scene will be little or even no DNA residue. Polygraph detection technology can help identify the authenticity of criminal activity. For example, the screenwriter of a drama film called Lie to Me shaped a human lie detector character who could recognize anyone’s lie. Dr. Carl Letterman, the hero in this film, could easily read the inner secrets of people through micro-expression recognition technology. However, in real life, the result of a lie detection is greatly affected by the subjective factors of testers, and testers can use anti-detection methods such as camouflage to greatly reduce the performance of the lie detection technology. Trace material evidence identification technology can be used to solve crimes by capturing trace material evidence at crime scenes, but the effectiveness of forensics by this technology largely depends on the integrality of fragmented evidence collected at crime scenes. In conclusion, different criminal investigation techniques have different technical weak points.

Thermal imaging technology is a kind of passive imaging technology [7,8]. Under some certain conditions, it can fully exert its technical advantages and form an advantage complementarity with other technologies to assist in the detection of difficult cases [9]. By using thermal imaging technology to collect images of the ground touched by the feet of the perpetrator, the curve of the decreasing temperature can be obtained, thus we can acknowledge the time when the perpetrator left through reverse inference. Compared with other criminal investigation technologies, the psychological factors of the testers have no impact on the performance of thermal imaging technology, and its requirements on hardware architecture and test environment are relatively lower. However, is has the disadvantage of being limited by time. That is, after the crime happens, when the residual heat on the ground gradually reaches the ambient temperature as time goes by, this technology will fail in a way. Therefore, for some unsolved cases, if we were able to obtain the heat sequence of the perpetrators’ feet left on the ground within definite time windows after they left the crime scenes, the remaining effective information could be captured, which would possibly be of great help to the detection at that time.

Far-infrared thermal imaging has been widely used in respiratory monitoring in telemedicine [10,11], obstacle detection [12,13] and pedestrian detection [14,15] in smart traffic, fault detection in smart industry [16,17], fire safety in smart cities [18,19], body temperature detection of pig in agriculture [20], etc. In criminal investigation, Pavlidis proposed that the facial image of a person could be obtained by thermal imaging technology, and then relevant analysis could be carried out to determine whether the person has fraudulent behavior [21]. Since then, thermal imaging technology has been commonly applied in security inspection [22,23]. Zalewski [24] proposed the use of thermal imaging technology for password cracking at the first time; on this basis, Mowery et al. [25] elaborated the principle of thermal imaging-based password cracking technology; Li et al [26]. established a physics model with the heat sequence image that could be used to crack the password, and then cracked the password through the inversion algorithm with an accuracy of 26% for the password cracking; Abdelrahman et al. [27] utilized the heat trace tracking technology to study the feasibility of cracking the unlock password and the unlock pattern of a mobile phone, and the results of research showed that even for repeated unlocking passwords, the cracking accuracy was higher than 72%. However, their article lacks an effective physical model to explain the cracking process. Kang et al. [28] pointed out that such phenomenon is like fire in a human’s hand, which can be used to understand some human behavior patterns.

The existing related literature largely focuses on how to crack codes through the residual heat left by hands. To the best of our knowledge, none of the reported techniques applies thermal imaging technology to measure thermal footprints (heat sequences left by foot on the ground) to inversely detect the time traces. Therefore, through the study of the thermal footprint models, we found the important relationship between the invisible residual temperature information on the ground and the departure time. Thermal footprint is a kind of criminal trace which will be easily ignored and hard to be eliminated quickly. Within a certain time window, the police can take advantage of the thermal footprints left at crime scenes to capture time information about the crime. If the time window is too long, the police cannot get much useful thermalfoot information. The faint heat traces can be used to infer the criminals’ target and escape route, which is important to narrow down the range of suspects. Also, we believe that, with the development of technology, thermal cameras are going cheaper and can be installed in more places, such as banks, supermarkets, some houses, and mobile terminals used by consumers. When crimes occur, thermal cameras can timely catch the heat traces without the help of the police. With further study, there can be some intelligent systems connected to thermal camera, which can automatically extract the ROIs of heat traces, outputting the departure time or other useful information. Thus, successfully applying thermal footprint technology will definitely provide valuable information for the police to break cases, bring more guarantees for people’s healthy life and social security and stability, cater to the major campaign against gang crime in the People’s Republic of China [29], and promote the development of smart public security in a certain sense.

The specific objectives of the current study are to (1) acquire the thermal sequence of feet prints and establish the thermalfoot model based on Newton’s Law of Cooling, (2) design validation experiments to evaluate the effectiveness of thermalfoot model on estimating departure time, (3) compare the performance of thermalfoot model with the performance of two kinds of subjective calculation methods on departure time reversal, and (4) discuss several factors that may affect the performance of thermalfoot model.

## 2. Materials and Methods

### 2.1. Framework of Thermalfoot

**Figure 1:**
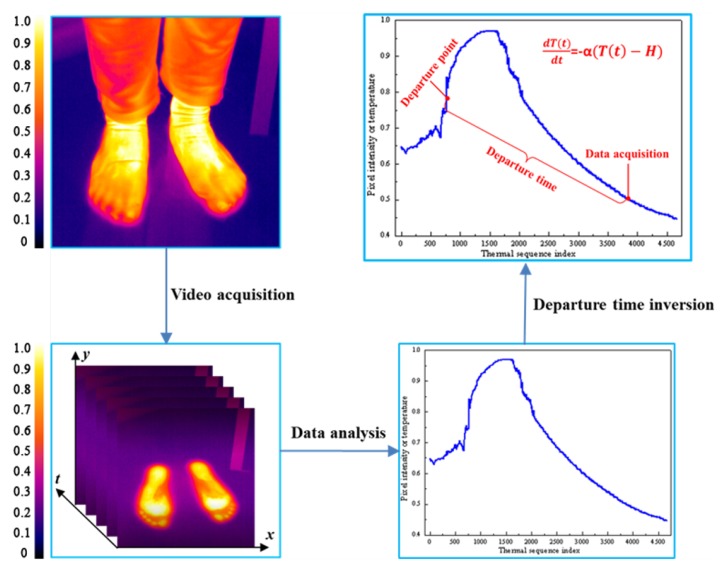
Scheme of departure time estimation. (The pixel values are normalized.)

As shown in Figure 1, when subjects were walking on the floor, owing to the temperature difference between the ground and human feet, thermal trace would be left on the floor. We define it as a “thermalfoot print”.

Thermal imaging is a passive imaging technique, and it can record the emitted energy from the objects whose absolute temperature is higher than zero without any external stimulation such as harmful radiation and illumination [30]. Considering the characteristic of far-infrared band, thermal imager is able to capture this thermal trace, so as to obtain a set of thermal sequences. The thermal sequence can be converted into one-dimensional thermalfoot print signal through preprocessing. According to Newton’s law of cooling, residual heat on the floor would gradually decrease with time, so the curve presented an exponentially decreasing tendency and finally remained basically unchanged. We obtained the model of time and temperature based on Newton’s law of cooling. If we have access to the thermalfoot print information within a certain time window, the departure time of subjects can be worked out through inverse analysis by using the model we gained.

**Figure 2:**
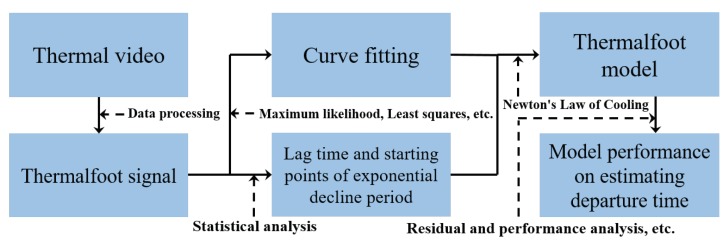
Workflow of the proposed algorithm.

The overall algorithm flow chart of this paper can be seen in Figure 2. Having gained thermal video by thermal imager, we obtained one-dimensional thermalfoot print signal by adopting a series of data processing methods. Later, two kinds of processing were carried out with thermalfoot print signal: (1) through qualitative analysis, we found that there were two unique characteristics, lag time and starting points of exponential decline period, existing in it. It was different from the existing literature [26]. In the practical application of model, lag time and starting points of exponential decline period are completely impossible to be accurately or precisely measured. Therefore, we must do a statistical analysis of these two variates to work out their possible values under some limitations based on prior knowledge; (2) during the exponential decline period, for the purpose of simplifying the problem, we changed non-convex optimization into convex optimization and assumed that the noise term conformed to the Gaussian distribution. Subsequently, the curve was fitted by the algorithm based on the Maximum likelihood method and the Least squares method to establish an exponentially decreasing relation formula of temperature and time. To evaluate the model performance on estimating the departure time, we adopted the evaluation method mainly focusing on residual and performance analysis.

### 2.2. Experimental Data Acquisition

We used a thermal imager (MAG 62, Magnity Electronics Co., Ltd., Shanghai, China) to obtain thermal videos of the experimental scene and after deleting abnormal data, we extracted more than 100 sets of thermal curves from thermal videos through image processing operations. The thermal camera has been corrected by the manufacturer (Magnity Electronics Co., Ltd., Shanghai, China) when leaving factory. The spectral range and thermal sensitivity of thermal imager are 7.5 to 14 μm and 0.5 ${}^{\circ }$C, respectively. The output thermal images are stored in BitMaP format. The temperature measurement range is from −20 ${}^{\circ }$C to 150 ${}^{\circ }$C with the temperature resolution of 1 ${}^{\circ }$C, indicating the reliability of the thermal imager. During the experiment, the participants stood on the floor with socks on their feet for a period of time. Right upon their leaving, we started to record videos over against the footprints traces by using the thermal camera, so as to obtain the heat sequence for subsequent analysis. The participants weighed between 45 and 80 kg and the ambient temperature ranged from 16 ${}^{\circ }$C to 28 ${}^{\circ }$C. We limited the floor material and the standing time of the subjects that the floor was a kind of ordinary wooden floor and the standing time ranged from 30 to 180 s, respectively. In our experiment, all the subjects wore socks, which is more in line with the actual application. Taking criminal condition into account, the perpetrators will not tend to wear shoes in order to prevent residues such as mud stains on the ground. In addition, bare feet can cause body fluids to remain on the ground. Therefore, wearing socks is the best option. As the socks are relatively thin, wearing socks or not does not affect the trend of the thermal curve. During the test, the real ambient temperature and the test time were obtained by thermometer and stopwatch respectively.

To reduce the noise caused by the factors like vibration in the process of experiment, the thermal imager was fixed on a tripod. The size of focal plane array of thermal imagery is 640 × 480 (corresponds to the spatial resolution of the camera) and the pixel pitch is 17 μm. The thermal imager communicated with a computer through a network cable. The collected thermal videos were finally sent to MATLAB R2018b (The Mathworks, Inc., Natick, MA, USA) for further analysis.

### 2.3. Preprocessing of Thermal Sequence Data

The thermal videos were first subjected to gray processing. Subsequently, we selected a certain square area within each footprint at the first frame after the feet left, and calculated the average pixel value in that area via the following equation, thus acquiring one-dimensional thermal footprint signals. The variation of frames were afterwards mapped to the changes of time based on the relationship between the frame and time.

(1) $$\bar{s}\left(x\right)=\frac{1}{n}\sum_{i,j\in N}s\left(i,j,x\right)$$

where *s*(*i*, *j*, *x*) is the pixel intensity of thermal image at pixel (*i*, *j*) and video frame x; *N* is the vector of pixel coordinates in Region of Interest (ROI) and n is its number.

## 3. Thermalfoot Model

The original temperature curve reflecting the temperature difference between the foot and the ground can be easily extracted via the simple image processing operation. However, our study is higher than the extraction of temperature difference curve. There are two challenges for our research: (1) find the general model that can describe the change laws of temperature curve. Because of the body weight, in the actual experimental process, we can observe the following fact: there are three different types of curves will be generated, bringing a lot of difficulties for us to establish the thermalfoot model; (2) in practical applications, due to the absence of prior knowledge, we can only use the limited observational data to fit the thermalfoot model to carry out departure time reversal. It is difficult to reverse the departure time using the limited observational data, and we adopt several methods to reduce the error.

### 3.1. Determination of Starting Points of Exponential Decline Period

To automatically find out the starting points of exponential decline period, procedures listed below were used.

To reduce the random noise, the fluctuation of instrument performance and ambient temperature, we used data smoothing processing for raw data $\left\{D_{k},k=1,2,...,n\right\}$, and the corresponding smooth curves $\left\{{\hat{D}}_{k},k=1,2,...,n\right\}$ were obtained. Smoothing filters have limitations in removing the abrupt points, which may affect the overall trend of the curve. To eliminate these abrupt points, the following two constraints are adopted.

Constraint (1): By analyzing a large number of curves, we found that the normalized brightness difference between two adjacent points will exceed 0.05. Therefore, abrupt points can be screened out by ensuring that the brightness difference between the current time point $x_{k,i}$, and the later time point $x_{k,i+1}$ does not exceed 0.05. For any time points $x_{k,i}$ in ${\hat{D}}_{k}$, the temperature values or the pixel intensities should obey the relationship below.

(2) $$|y_{k,i}-y_{k,i+1}|<0.05,i=1,2,...,N_{k}$$

where *i* is the time index, and $N_{k}$ is the total number of points on the $K_{th}$ thermal curve.

The first constraint can help us remove a large number of abrupt points. Let the time value set of the point set in the local domain obtained by constraint (1) as $A=\{\alpha_{k,i^{{}^{\prime }}},k=1,2,...,n\}$, where $i^{\prime }\in \left\{1,2,...,N_{k}\right\}$. Subsequently, constraint (2) was used to continue looking for starting point of exponential decline period. To prevent getting a local drop point, we compared each point with the average of the next 100 points: if the brightness value of this point is greater than or equal to the average brightness value of the next 100 points, the curve is considered to have a downward trend at this point.

Constraint (2): to make the temperature value $y_{k,i}$ corresponding to the time point $x_{k,i}$ greater than or equal to the average temperature of the next 100 points, the following relationship should be satisfied.

(3) $$y_{k,i}\geq \frac{1}{100}\sum_{m=1}^{100}y_{k,i+m}$$

Now, we obtain the time point set containing the targeted starting point of exponential decline period $B=\{\beta_{k,i^{\prime \prime }},k=1,2,...,n\}$, where $i^{\prime \prime }\in \left\{1,2,...,N_{k}\right\}$.

To find the time point set, Γ which simultaneously meets constraints (1) and (2), the intersection of *A* and *B* is calculated.

(4) $$\Gamma =A\cap B=\left\{\gamma_{k,t},k=1,2,...,n\right\},t\in \left\{1,2,...,N_{k}\right\}$$

The first time point in Γ with minimum time index was regarded as the targeted starting point of exponential decline period. Let assume that the set *C* includes the alternative time points.

(5) ρ=minC

$\gamma_{k,\rho }$ is the starting point of exponential decline period for the $k_{th}$ smooth thermal curve ${\hat{D}}_{k}$.

### 3.2. Determination of Departure Points

**Figure 3:**
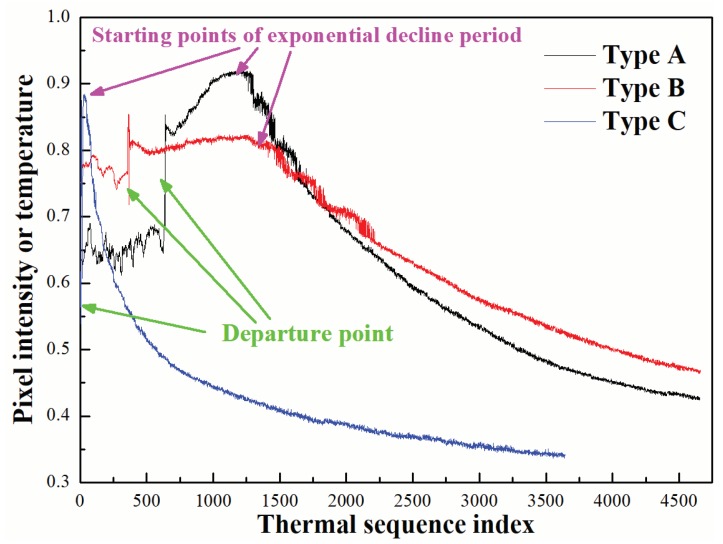
Three typical thermal curves extracted from raw thermal videos.

As we can see in Figure 3, an abrupt point occurs on the thermal curve when the subject leaves. The pixel intensity or temperature changes dramatically around the abrupt point. Based on this characteristic, we design an algorithm which can obtain the information about such change. To reduce the disturbance of other abrupt points, especially the points around the decline period, we only analyze the points before the starting point of exponential decline period.

In Section 3.1, we have obtained the starting points of exponential decline period for the $k_{th}$ smoothed thermal curve ${\hat{D}}_{k}:\gamma_{k,\rho }$. Subsequently, we choose temperature values $\left\{y_{k,1},y_{k,2},\ldots ,y_{k,m}\right\}$ of all points before the time point $\gamma_{k,\rho }$ and assume

(6) $$Y_{k,i}=\left[\begin{array}{cccc} y_{k,1} & y_{k,2} & ... & y_{k,m-1} \end{array}\right]\;$$

(7) $$Y_{k,j}=\left[\begin{array}{cccc} y_{k,2} & y_{k,3} & ... & y_{k,m} \end{array}\right]\;$$

The value of $|Y_{k,i}-Y_{k,j}|$ is used to reflect the degree of temperature difference between the adjacent time point. The higher is the degree of temperature difference, the bigger is the value of $|Y_{k,i}-Y_{k,j}|$. To find the biggest difference value $d_{k,q}$, the following relationship should be satisfied,

(8) $${\hat{y}}_{k,q}=max(|Y_{k,i}-Y_{k,j}\left|\right),1\leq q\leq m-1$$

where $d_{k,q}$ is the maximum temperature difference for the $k_{th}$ smoothed thermal curve ${\hat{D}}_{k}$. Therefore, the targeted temperature value $y_{k,q}$ corresponding to $n_{k,q+1}$ should satisfy the relationship

(9) $$y_{k,q}={\hat{y}}_{k,q+1}$$

where $y_{k,q}$ is the most reasonable temperature value of departure time. According to thermalfoot model, we finally get the departure time point $n_{k,q+1}$ for the $k_{th}$ smoothed thermal curve ${\hat{D}}_{k}$.

### 3.3. Establishment of Thermal Footprint Model

After the subject left the floor, the temperature where the subject stood is higher than the atmosphere. According to Newton’s law of cooling, the surface temperature of the floor where tester stood begins decreasing, and the rate of change is in proportional to the difference between the surface temperature and steady temperature. $H_{i}\left(t\right)$ is the observed temperature, $Hs_{i}\left(t\right)$is the steady temperature. λ is proportional coefficient, which is a constant. Therefore the differential equation is as follows,

(10) $$\frac{dH_{i}\left(t\right)}{dt}=-\lambda \left(H_{i}\left(t\right)\right)-{Hs}_{i}).$$

We assume

(11) $$H^{\prime }\left(t\right)=H_{i}\left(t\right)-Hs_{i}.$$

Subsequently, we get

(12) $$\frac{dH^{\prime }\left(t\right)}{dt}=\frac{d(H_{i}\left(t\right)-Hs_{i})}{dt}=\frac{dH_{i}\left(t\right)}{dt}-\frac{dHs_{i}}{dt}=\frac{dH_{i}\left(t\right)}{dt}-0=\frac{dH_{i}\left(t\right)}{dt}=-\lambda \left(H_{i}\left(t\right)\right)-{Hs}_{i})=-\lambda H^{\prime }\left(t\right),$$

Therefore,

(13) $$\frac{dH^{\prime }\left(t\right)}{dt}=-\lambda H^{\prime }\left(t\right).$$

We solve (13)

(14) $$H^{\prime }\left(t\right)=H_{0}^{{}^{\prime }}*e^{-\lambda t},$$

where $H_{0}^{{}^{\prime }}=H^{\prime }\left(0\right)=H_{i}\left(0\right)-Hs_{i}$. By combining (11) and (14), we obtain the temperature change with time:

(15) $$H_{i}\left(t\right)=\left(H_{0}-Hs_{i}\right)*e^{-\lambda t}+Hs_{i}.$$

The discrete form of (15) is

(16) $$H_{i,k}=\left(H_{i},1-Hs_{i}\right)*e^{-\lambda^{\prime }\Delta t_{k}}+Hs_{i},$$

where k=1,2,...,N is the time index, $\Delta t_{k}=t_{k}-t_{k-1}$ is time period. Considering one frame of thermal camera equals to 25 seconds, we assume $\Delta t_{k}$ are close to zero. The temperature change rate is

(17) $$\frac{dH_{i}\left(t\right)}{dt}=lim_{\Delta t_{k}\to 0}\frac{H_{i,k}-H_{i,k-1}}{\Delta t_{k}}\approx \frac{H_{i,k}-H_{i,k-1}}{\Delta t_{k}}.$$

By combining (10) and (17), we obtain the relationship between adjoining time index of temperature:

(18) $$\frac{H_{i,k}-H_{i,k-1}}{\Delta t_{k}}=-\lambda \left(H_{i}\left(t\right)\right)-{Hs}_{i}),$$

Therefore,

(19) $$H_{i,k}-H_{i,k-1}=-\lambda *\Delta t_{k}\left(H_{i}\left(t\right)-Hs_{i}\right).$$

Taking noise caused by thermal camera into account, we define

(20) $$H_{i}\left(t\right)=h_{i}\left(k|\theta_{i}\right)+\varepsilon_{i}\left(t\right),$$

where $H_{i}\left(t\right)$ is the observed temperature, and $h_{i}\left(k|\theta \right)$ is the actual temperature. $\varepsilon_{i}\left(t\right)$ is the noise caused by thermal camera. The discrete form of (20) is

(21) $$H_{i,k}=h_{i}\left(k|\theta \right)+\varepsilon_{i,k},$$

where $H_{i}$=$\left\{H_{i,k},k=1,2,...,N\right\}$ is sample data of observed temperature.

To obtain the relationship between actual temperature and time, we need to further study function $h_{i}\left(k|\theta \right)$. Except for some special processing methods [31], the conventional method in most papers is to assume that the noise obey Gaussian distribution [32,33,34,35]. We assume noise $\varepsilon_{i,k}$ obeys Gaussian distribution. Therefore, we can define the conditional likelihood function of $H_{i,k}$:

(22) $$p\left(H_{i,k}|\theta_{i}\right)=\frac{1}{\sqrt{2\pi \sigma_{i}}}*e^{\frac{{\left[H_{i,k}-h_{i}\left(k|\theta_{i}\right)\right]}^{2}}{\sigma_{i}^{2}}},$$

where $\sigma^{2}=\frac{1}{N}\sum_{i=1}^{N}{\left(\bar{\varepsilon_{i}}-\varepsilon_{i}\right)}^{2}$,$\bar{\varepsilon }=\frac{1}{N}\sum_{i=1}^{N}$. We find parameter θ determines the function $h_{i}\left(k|\theta \right)$, and θ is estimated by maximizing likelihood function:

(23) $$\hat{\theta }=d\left(H_{i}\right)=d\left(H_{i,1},H_{i,2},...,H_{i,N}\right)=\underset{\theta_{i}}{\arg\max}P\left(H_{i}|\theta_{i}\right),$$

where $P(H_{i}|\theta_{i})$ is the k=likelihood function for $H_{i}$:

(24) $$P\left(H_{i}|\theta_{i}\right)=\prod_{i=1}^{N}p\left(H_{i,k}|\theta_{i}\right)={\frac{1}{{\left(2\pi \sigma_{i}\right)}^{2}}}^{\frac{N}{2}}\prod_{i=1}^{N}e^{-\frac{{\left[H_{i,k}-h_{i}\left(k|\theta_{i}\right)\right]}^{2}}{2\sigma_{i}^{2}}}.$$

According to (15), we get

(25) $$h_{i}\left(k|\theta_{i}\right)=\left(H_{i,1}-hs_{i}\right)e^{-\lambda_{i}k}+hs_{i},$$

where $\theta =\{hs_{i},\lambda_{i}\}$ are the parameters we need to estimate. Therefore, we maximize the likelihood function of $P(H_{i}|\theta_{i})$:

(26) $$\hat{hs_{i}},\hat{\lambda_{i}}=\underset{hs_{i},\lambda_{i}}{\arg\max}P\left(H_{i}|hs_{i},\lambda_{i}\right).$$

According to (19), we can find $H_{i,k}$ and $H_{i,k-1}$ has linear relation. Therefore, the optimization problem is convex:

(27) $$H_{i,k}-H_{i,k-1}=\lambda_{i}\left(H_{i,k}-hs_{i}\right)+\delta_{i,k},$$

where we assume $\delta_{i,k}$ is an error term caused by noise, which should obey Gaussian distribution. According to (22), we get

(28) $$p\left(H_{i,k}|\theta_{i}\right)=\frac{1}{\sqrt{2\pi \sigma_{i}}}*e^{\frac{{\left[H_{i,k}-H_{i,k-1}-\lambda_{i}\left(H_{i,k}-hs_{i}\right)\right]}^{2}}{\sigma_{i}^{2}}}.$$

Therefore, especially according to (24), we can obtain the likelihood function of $H_{i}$=$\left\{H_{i,k},k=1,2,...,N\right\}$:

(29) $$P\left(H_{i}|\theta_{i}\right)=\prod_{i=1}^{N}p\left(H_{i,k}|\theta_{i}\right)={\frac{1}{{\left(2\pi \sigma_{i}\right)}^{2}}}^{\frac{N}{2}}\prod_{i=1}^{N}e^{-}\frac{{\left[H_{i,k}-H_{i,k-1}-\lambda_{i}\left(H_{i,k}-hs_{i}\right)\right]}^{2}}{2\sigma_{i}^{2}}.$$

Log likelihood function of (29) is

(30) $$logP\left(H_{i}|\theta_{i}\right)=\prod_{i=1}^{N}logp\left(H_{i,k}|\theta_{i}\right).$$

(30) is proportional to $\sum_{i=1}^{N}-\frac{{\left[H_{i,k}-H_{i,k-1}-\lambda_{i}\left(H_{i,k}-hs_{i}\right)\right]}^{2}}{2\sigma_{i}^{2}}$. Therefore, we can obtain

(31) $$\hat{hs_{i}},\hat{\lambda_{i}}=\underset{hs_{i},\lambda_{i}}{\arg\min}\sum_{i=1}^{N}{\left[H_{i,k}-H_{i,k-1}-\lambda_{i}\left(H_{i,k}-hs_{i}\right)\right]}^{2}.$$

We assume that if we already obtain estimated parameters $\hat{hs_{i}},\hat{\lambda_{i}}$, so we can obtain the new temperature samples ${H_{i}}^{{}^{\prime }}=\left\{{H_{i,k}}^{{}^{\prime }},k=1,2,...,N\right\}$ by using thermalfoot model. Now we need to use Least Mean Square to solve the optimization problem. We define

(32) $$Q_{i}=\left[\begin{array}{cc} H_{i,1} & -1\\ H_{i,2} & -1\\ ... & ...\\ H_{i,N-1} & -1 \end{array}\right]\;.$$

(33) $$q_{i}={\left[\begin{array}{cccc} H_{i,2}-H_{i,1} & H_{i,3}-H_{i,2} & ... & H_{i,N}-H_{i,N-1} \end{array}\right]\;}^{T}.$$

(34) $$\chi_{i}={\left[\begin{array}{cc} \lambda_{i} & \lambda hs_{i} \end{array}\right]\;}^{T}.$$

Therefore, we can obtain

(35) $$\hat{\chi_{i}}=\underset{\chi_{i}}{\arg\min}{\left|\right|Q_{i}\chi_{i}-q_{i}\left|\right|}_{2}^{2}.$$

By solving (35), we can get

(36) $$\hat{\chi_{i}}={\left({Q_{i}}^{T}Q_{i}\right)}^{-1}Q_{i}^{T}q_{i}.$$

Now, we can obtain the relationship between actual temperature and time according to (25).

### 3.4. Evaluation of Thermal Footprint Model

The goodness of fit of thermalfoot model is evaluated using the following equation: we consider that $\bar{x_{N}}$ is the mean value of a set of original temperature sample data $\left\{x_{1},x_{2},\ldots ,x_{N}\right\}$. According to the thermalfoot model, we obtain the new temperature sample data $\left\{y_{1},y_{2},\ldots ,y_{N}\right\}$, the mean value of which is $\bar{y_{N}}$. We calculate the correlation coefficient r:

(37) $$r=\frac{\sum_{i=1}^{N}\left(x-\bar{x_{i}}\right)\left(y-\bar{y_{i}}\right)}{\sqrt{\sum_{i=1}^{N}{\left(x-\bar{x_{i}}\right)}^{2}}\sqrt{\sum_{i=1}^{N}{\left(y-\bar{y_{i}}\right)}^{2}}}$$

where the range of r is from −1 to 1. When r ranges from −1 to 0, the smaller r is, the bigger the correlation is; when r ranges from 0 to 1, the bigger r is, the bigger the correlation is.

By observing the thermalfoot curve, we can find three factors accounting for model performance: the calculation of lag time, the selection of starting point, and the goodness of fit of thermalfoot model. As to the lag time and starting points of the exponential decline period, we conduct a statistical analysis based on 50 datasets, obtaining their normal distributions respectively. Subsequently, the mean values of these two normal distributions are selected as constant terms for departure time estimation under the condition where the atmosphere temperature ranges from 16 ${}^{\circ }$C to 28 ${}^{\circ }$C.

In the experiment of estimating the departure time using thermalfoot model, we consider the following two situations:

(1) Assume that the starting point of the exponential decline period is known; we can obtain the departure time $\left\{\alpha_{1},\alpha_{2},\ldots ,\alpha_{N}\right\}$ according to thermalfoot model. As for 50 datasets, four different capture time points viz. one-third, one-half, two-thirds, and three-fourths are chosen, and the corresponding estimated departure time $\left\{\beta_{1},\beta_{2},\ldots ,\beta_{N}\right\}$ can be derived by using thermalfoot models. The residual is applied to assess the performance of thermalfoot model for the departure time reversal:

(38) $$\Delta_{i}=\alpha_{i}-\beta_{i},i-1,2,...,N$$

(2) Use the departure time obtained by stopwatch as the ground-truth departure time: under the above situation, the ground-truth departure time is calculated from the thermalfoot model; however, it is unreasonable for practical use. As a consequence, we set the departure time $\left\{\gamma_{1},\gamma_{2},\ldots ,\gamma_{N}\right\}$ obtained by stopwatch as the ground truth. In the same way, four capture time points viz. one-third, one-half, two-thirds, and three-fourths are selected, and the estimated departure time $\left\{\beta_{1},\beta_{2},\ldots ,\beta_{N}\right\}$ can be obtained by using thermalfoot model. The residual is utilized to assess the thermalfoot model performance for the departure time reversal:

(39) $$\Delta^{\prime }=\gamma_{i}-\beta_{i},i=1,2,...,N$$

Nevertheless, in practical application, we are more concerned about the concept of time interval. For example, in most cases, we just want to know the approximate time someone left, not the exact time point. Therefore, we count the number of the estimated departure time falling within the acceptable time interval, and then calculate the accuracy. These estimated departure times are obtained by thermalfoot model at different capture time points (one-third, one-half, two-thirds, three-fourths, fourth-fifths, and five-sixths). The specific steps to calculate the model’s reverse accuracy are as follows:

(i) Calculate the acceptable time intervals: the acceptable time intervals are estimated by thermalfoot model. Six sets of local curves are obtained at different capture time points (one-third, one-half, two-thirds, three-fourths, fourth-fifths, and five-sixths), and each set has 107 local curves. Subsequently, the values of upper boundary and lower boundary of confidence intervals of starting points and lag time in their normal distribution curves are combined to calculate the minimum and maximum temperatures at the starting points. Based on these temperature intervals, the acceptable time intervals can be obtained via the thermalfoot model. There are the acceptable time intervals for each local curve set.

(ii) Calculate the model’s reverse accuracy: we define a function sgn(τ):

(40) $$sgn\left(\tau \right)=\left\{\begin{array}{c} 1,\;if\;\tau \;is\;in\;the\;time\;interval\\ 0,\;if\;\tau \;is\;out\;the\;time\;interval \end{array}\right.$$

where τ is the actual departure time and $\left\{\tau_{1},\tau_{2},\ldots ,\tau_{N}\right\}$ is its set. The accuracy is computed as follows.

(41) $$\rho =\frac{\sum_{i=1}^{N}sgn\left(\tau_{i}\right)}{N},i=1,2,...,N$$

To further compare the effectiveness of thermalfoot model for the departure time reversal in actual application, we use two subjective calculation methods to make the comparison with thermalfoot model calculation method. As for six local curve sets, two types of subjective evaluation approaches are adopted. Subjective calculation 1: we directly estimate the departure time according to obtained local curves. Subjective calculation 2: we utilize auxiliary means such as a ruler to estimate the departure time based on obtained local curves.

Similar to thermalfoot model calculation, we use the Formulae (40) and (41) to calculate the accuracy obtained by subjective calculation approaches.

## 4. Results

### 4.1. Qualitative Analysis of Thermal Sequence Curves

Having thoroughly analyzed all the obtained curves, we divided them into three types (Figure 3):

Type A (slowly rising): when human feet left the ground, the curve slowly rose to the highest point, then decreased exponentially, and finally reached a steady state.

Type B (stationary): when human feet left the ground, the curve remained in a state of extremely slight fluctuation until the curve decreased exponentially, and finally reached a steady condition.

Type C (steeply rising): when human feet left the ground, the curve rose sharply and rapidly to the peak, then immediately decreased exponentially, and finally achieved a steady state.

We defined the time when human feet left the ground as departure point. Specifically, for type A and C, the point of highest pixel intensity was defined as starting point of exponential decline period.

Based on different features of the above curves, we analyzed the reasons why they demonstrated three different states between departure points and starting points of exponential decline period.

Type A (slowly rising): we suspect that human feet applied force to the ground as they left, making heat rise of the ground, which led to a slowly rising process on the curve. In addition, sensor delay (i.e., the sensor switching may lag behind the actual image switching) would also cause a slowly rising process on the curve after feet left.

Type B (stationary): after feet left the ground, the curve went through a slight fluctuation, then according to Newton’s law of cooling, decreased exponentially and finally achieved a steady condition. It is an ideal curve.

Type C (steeply rising): we assume that short measuring time and sharp pixel changes made the curve steeply rise after feet left the ground.

On the basis of Newton’s law of cooling, the temperature of the ground would lose heat constantly. Each curve would present an exponentially decreasing tendency after a time point, and reach a stable state in the end (i.e., being basically the same as ambient temperature).

### 4.2. Statistical Analysis of Lag Time and Starting Point of Exponential Decline Period

**Figure 4:**
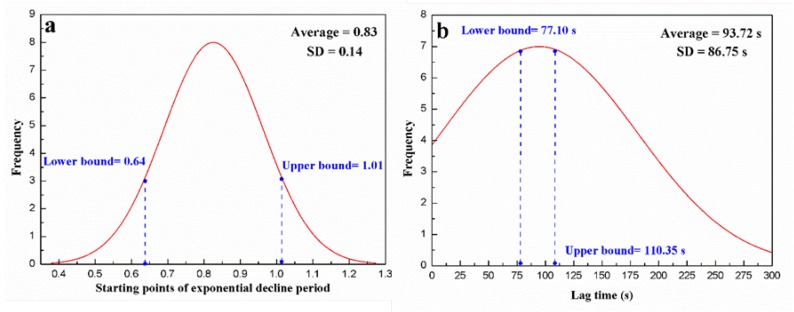
Normal distributions of starting points of exponential decline period (**a**) and lag time (**b**) in our dataset.

According to the algorithms in Section 3.1 and Section 3.2, we obtained 107 sets of starting points and lag times. The normalized statistical indicators (average, ±standard deviation) are 0.83, ±0.14 and 93.72 s, ±86.75 s for the starting point (Figure 4a) and lag time (Figure 4b), respectively. Under 95% confidence interval, [0.64, 1.01] and [77.10 s, 110.35 s] are, respectively, selected as the estimated ranges for the starting point and lag time when the environment temperature ranges from 16 ${}^{\circ }$C to 28 ${}^{\circ }$C. These two estimated intervals will be used for the subsequent departure time estimation.

### 4.3. Analysis of Performance of Model Fitting

#### 4.3.1. Performance of Thermalfoot Model Fitting

**Figure 5:**
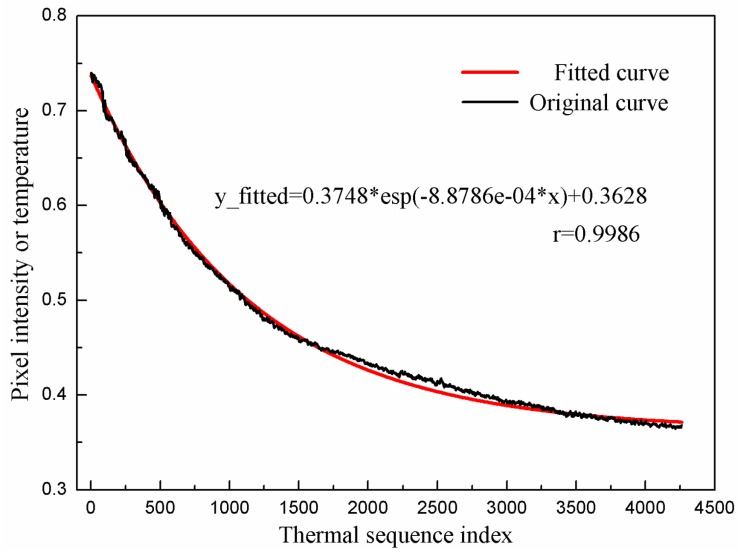
One typical fitting result for exponential decline phase using Newton’s Law of Cooling.

We constructed a thermalfoot model using a set of raw data (from the starting point to the steady state), and the fitting result is shown in Figure 5. Based on the physical significance of thermalfoot model, in the analytical expression, 0.3748 is the difference between the temperature at the starting point of exponential decline period and that at the steady state; the coefficient before *x* is determined by Newton’s law of cooling; 0.3628 is the temperature value for the steady state. Note that all temperature values are normalized. The correlation coefficient between the original temperature value and the fitted temperature value is 0.9986, indicating the obtained thermalfoot model can basically reflect the variation of original data. The fitting results of the other curves in the data set are also satisfactory.

#### 4.3.2. Estimation of Departure Time

**Figure 6:**
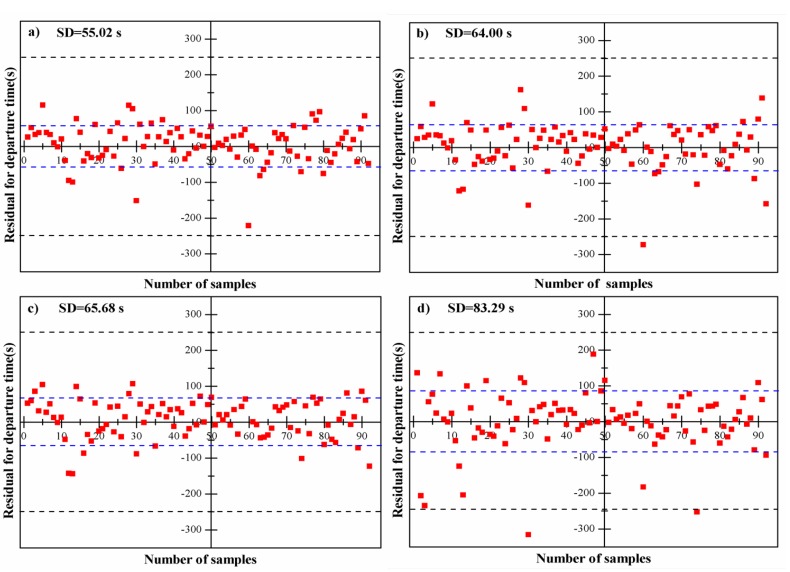
Residual for departure time at different points (*N* = 92) under the situation where the ground-truth departure time estimated by thermal footprint model: (**a**) one-third point, (**b**) one-half point, (**c**) two-thirds point, and (**d**) three-fourths point. (A total of 15 points with large deviations (>350 s) were eliminated).

Two cases were considered for residual analysis of departure time estimation:

(1) The starting points estimated by thermal footprint model: In this case, we assume that all data from the starting point of exponential decline period to the steady state are known and used for modeling. The ground-truth departure time was estimated by thermalfoot model. Subsequently, with respect to each thermal curve, four estimated departure times derived from four different capture time points viz. one-third, one-half, two-thirds, and three-fourths, were obtained using thermalfoot model. The residual analysis was carried out for evaluating the performance of thermalfoot model (Figure 6). We regarded data points whose residual value was beyond 350 s as outliers, and exclude these data when doing residual graph. A total of 15 points were eliminated. As shown in Figure 6, we can observe that almost all residuals randomly disperse around the line of perfect estimation (residual = 0 s) and are located within the upper boundary line of 250 s and the lower boundary line of −250 s, suggesting the performance of thermalfoot model is satisfactory. The standard deviation of departure time were 55.02 s, 64.00 s, 65.68 s, and 83.29 s for capture time of one-third, one-half, two-thirds, and three-fourths, respectively. This indicates that the performance of model will decrease as information we can use is reduced.

There is a serious problem in this case that the thermalfoot model was established based on all data, however, in practical applications, data before the capture time point are unknown. Case 2 meets the actual application conditions.

**Figure 7:**
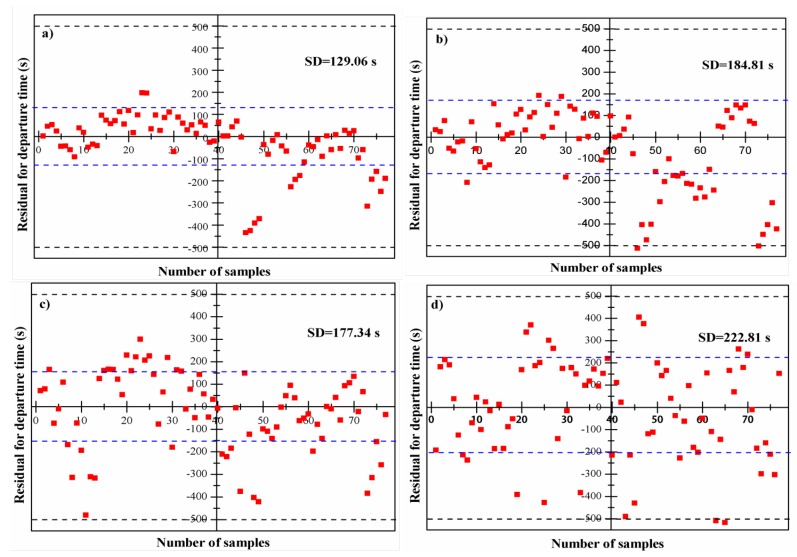
Residual for departure time at different points (*N* = 77) under the situation where the starting points of exponential decline period are estimated by statistical analysis: (**a**) one-third point, (**b**) one-half point, (**c**) two-thirds point, and (**d**) three-fourths point. (A total of 30 points with large deviations (>515 s) were eliminated).

(2) The starting points estimated by statistical analysis: in this case, we assume the starting points of exponential decline period follow the Gaussian distribution and fluctuate within a certain region under a certain ambient temperature. Therefore, an average value derived from the fitted Gaussian distribution was used for modeling. The ground-truth departure time was recorded by stopwatch. Then, in terms of each thermal curve, four estimated departure times derived from four different capture time points viz. one-third, one-half, two-thirds, and three-fourths were obtained using thermalfoot model. The residual analysis was conducted for evaluating the performance of thermalfoot model (Figure 7). We considered residual points whose value was greater than 550 s as outliers. A total of 30 outliers were eliminated when doing residual graphs. What we can observe from Figure 7 is that, almost all residuals randomly disperse around the line of perfect estimation (residual = 0 s) and are located within the upper boundary line of +500 s and the lower boundary line of −500 s. The performance of thermalfoot model is not as satisfactory as that in case 1, but it is still accepted, considering that case 2 is more actual than case 1. The standard deviation of departure time were 129.06 s, 184.81 s, 177.34 s, and 222.81 s for capture time of one-third, one-half, two-thirds, and three-fourths, respectively. This also demonstrated that, the performance of model would decrease as information we can make use of reduced.

#### 4.3.3. Accuracy of Estimating Departure Time at the Different Capture Time Points

**Figure 8:**
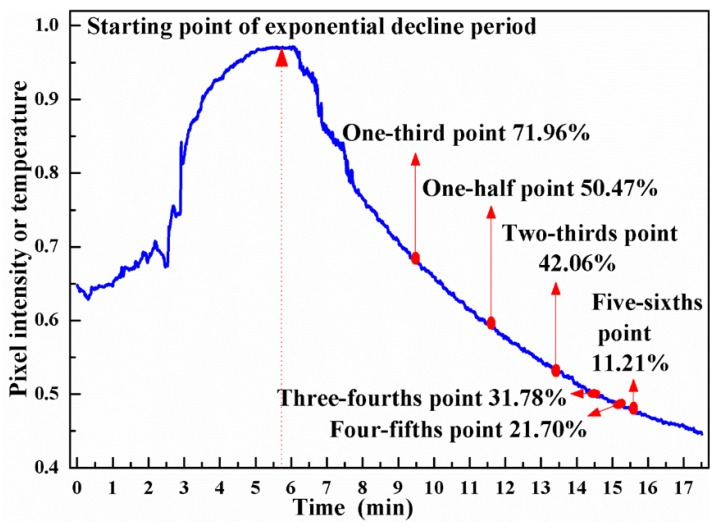
Accuracy of estimating departure time at the different capture time points.

The accuracy of estimating departure time at different capture time points (one-third point, one-half point, two-thirds point, three-fourths point, four-fifths point, and five-sixths point) is calculated using the thermalfoot model (Figure 8). As the curve approaches steady state, the accuracy of thermalfoot model for estimating departure time decreases from 71.96% to 11.21%. This phenomenon demonstrates that, for each thermalfoot model, there must be a failure point. The thermalfoot model will be unable to infer the departure time at capture time points behind the failure point.

### 4.4. Comparison with Subjective Calculation Method

To measure the effectiveness of using thermalfoot model to estimate the departure time, we compare the thermalfoot model calculation method with two subjective calculation methods, obtaining the results in Table 1. The accuracy obtained by three methods shows the same pattern, that is, the accuracy decreases as the information we can get from the thermalfoot curve reduces. As we can observe in Table 1, the subjective calculation${}^{2}$ outperforms the subjective calculation${}^{1}$. Nonetheless, as the information we can use is reduced, the difference between these two methods becomes small. In terms of three different capture time points—one-third, one-half, and two-thirds—the accuracy obtained by the thermalfoot calculation is all higher than that obtained by two subjective calculation methods. With regard to other capture time points viz. three-fourths, four-fifths and five-sixths, the accuracy obtained by the three methods is relatively close to each other. When the curve we choose approaches the starting point of the exponential decline period, the accuracy obtained by thermalfoot model calculation is relatively good (the accuracies are 71.96%, 50.47% and 42.06% for one-third, one-half and two-thirds, respectively.). The subjective calculation${}^{2}$ and the thermalfoot model calculation have similar results when the curve we choose approaches the steady state, where the local information is too little for us to estimate the overall trend. Therefore, it is demonstrated that the thermalfoot model is effective in actual applications.

In practice, if we can obtain thermalfoot information in a short time window, the thermalfoot model could be utilized to estimate the departure time; if we cannot, in other words, the thermalfoot information we obtain is not enough for us to only use thermalfoot model to solve the problem, and we can try to conduct a subjective calculation by using auxiliary means (such as ruler) on the basis of the thermalfoot model calculation. According to our experiments, only by doing so can we get a more satisfying result. Although the witness might know that the perpetrators just left just a little while ago, he or she could fail to know a more specific and accurate departure time. To speak of, the subjective calculation is finished by who participates in the study, more expert than others in some extend. Therefore, the thermalfoot model calculation method is better than a subjective calculation of a witness. Our methodology is effective when applied in actual condition.

## 5. Discussion

### 5.1. About "Outlier"

The outlier is in quotation marks because it was caused by the failure of model backstepping rather than instrumental measurement error. As analyzed in Section 3.4, we have known that the calculation of lag time and the selection of starting point have an effect on the result of model estimation. Therefore, how to determine the values of lag time and starting point of exponential decline period is the biggest challenge in the research of detecting departure time inversely by thermal foot model. Lag time means the period from people’s feet leave the floor to the thermal curve starts to decrease. Starting point of exponential decline period means the point when the thermalfoot curve begins to decrease. In actual application, without the help of other technologies, we cannot directly obtain the values of these two parameters. To solve this problem, as shown in Section 4.2, we made an assumption that within a certain temperature range, the lag time and the starting point of exponential decline period followed normal distribution, and their values all fluctuated within a certain region. Therefore, we did the analysis below. In the case of the assumption mentioned above, the backstepping results of model undoubtedly had a lot of errors. Based on the known starting point, 15 abnormal data were deleted in this study, and the remaining 92 data were used for residual analysis. On the account of the predicted starting point, we deleted 30 abnormal data and used the remaining 77 data for residual analysis. Abnormal data, which occurred in the situation of known starting point, was included in the 15 abnormal data, which were from the situation of predicted point. The situations where the starting points were predicted were close to the actual situations. Table 2 shows the outlier ratio of two situations: known starting point and predicted starting point under ambient temperatures of 16 ${}^{\circ }$C and 28 ${}^{\circ }$C. We found that the outlier ratios at two temperatures were basically the same if starting point was known (14.29% versus 13.85%). However, under the circumstance of predicted starting point, the outlier ratio at 16 ${}^{\circ }$C was lower than that at 28 ${}^{\circ }$C (26.29% versus 29.23%). It was possibly because the more ambient temperature was closed to the average temperature of the healthy human feet (27–30 ${}^{\circ }$C) [36], the faster the thermal curve reached the stable state. Based on that, less priori knowledge for building thermalfoot model could be acquired, which in turn resulted in more outliers.

### 5.2. Discussion of ROI Selection

**Figure 9:**
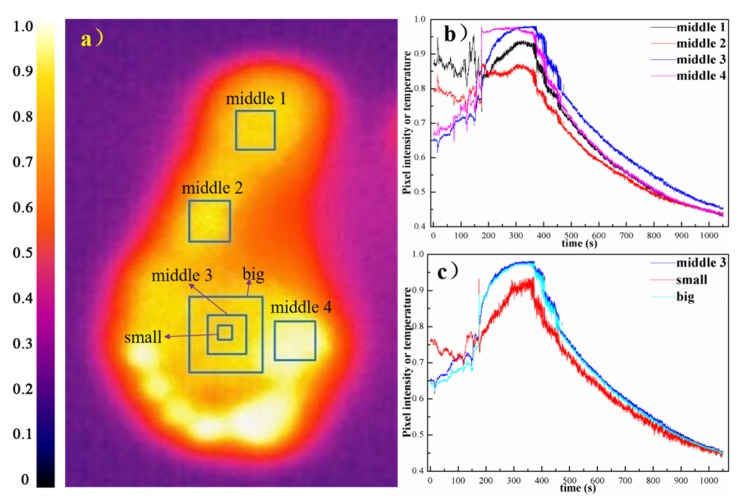
Influence of ROI selection on generation of thermal curve: (**a**) possible ROI selection in the sole of foot; (**b**) thermal curves extracted from different ROIs; and (**c**) thermal curves extracted from the same ROI with various sizes. (The pixel values are normalized)

In this study, the ROIs were randomly selected in the process of pretreatment, and we found that the shape of the curve was greatly influenced by different areas within the soles of feet and different sizes of ROIs, which is graphically presented in Figure 9.

The influence on thermal curve by the selection of ROI: when the sizes of ROIs were the same but locations were not (e.g., four areas of middle1, middle2, middle3, and middle4 in Figure 9), by comparing four curves, we found that there were differences between the maximum temperature values in different parts. Within the four RIOs extracted with same size, the area representing the root of big toe (i.e., middle4) demonstrated the highest temperature. Meanwhile, in the thermalfoot pattern, the temperature on the front sole of the foot was higher than that on the heel of the foot, and lower temperature might occur in the arch area. It was inconsistent with the results of previous literature [37]. The existing literature shows that the middle of the foot, i.e., the arch area, should have the highest temperature among other areas on the foot. We think that possibly it was the incomplete contact with the ground that caused the difference of the temperature distribution on the sole of the foot. As an air gap would be formed then between the foot and the ground because of that, thus thermal radiation and thermal convection would replace thermal conduction as the main methods of heat transfer, which resulted in a reduction in heat transfer ability [38]. Therefore, the temperature of arch area on the foot was lower than other areas.

Besides, there was small discrepancy in the noise of the four curves. By comparing the decent parts of curves, we found that the slope of the decent parts on the curves obtained by different ROIs were discrepant. We made a conjecture that the initial temperature of the selected areas and the slope of the decent curve might have a positive relationship to some extent.

The influence on thermal curve caused by the sizes of ROIs: when the location of the selected ROI was similar, but size was not (e.g., three areas of middle3, small, big in Figure 9), comparing the curves, it was plain to see that the curves of middle3 and big areas were basically in accordance with each other, but the temperature value of small area was lower among others on the whole with a more obvious noise. Verified by graphing other additional data (data not shown), we suspected that there was a negative relationship between the size of the selected area and noise to some degree, meaning that the smaller the selected area was, the bigger its noise was.

Generally, although there were differences between individual curves, the overall trend of curves was nearly the same. The departure points of the six curves concentrated in the time interval of 175 s to 200 s, and the starting points of the exponential decline period also intensively appeared in the time interval of 325 s to 375 s.

### 5.3. Influence of Background Radiation

**Figure 10:**
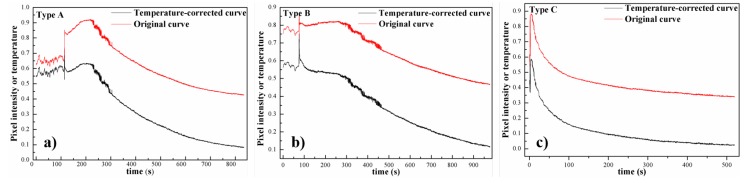
Three original thermal curves and their temperature-corrected curves: (**a**–**c**) are types A–C, respectively.

We consider the influence of background radiation on the thermalfoot curve, and the temperature-corrected curves for types A–C are shown in Figure 10. As shown in Figure 10, we can observe that the trend of temperature-corrected curves is similar to that of original curves. As a consequence, we can ignore the influence of thermal radiation of floor. In addition, the atmospheric temperature of the thermal footprints is constant and we think that whether to consider atmospheric temperature or not makes no difference.

### 5.4. Influence of Capture Time Point

According to the above analysis, thermal curve decreased exponentially with time at first, and gradually became stable after a certain period of heat exchange (including heat conduction, heat convection, and heat radiation). We found that the temperature at the stable state of each curve was not a certain value, which kept near but was not equal to the ambient temperature. In general condition, the higher the starting point of exponential decline period, the higher the temperature value of the stable state. Moreover, the closer the capture time point is to the steady state, the less accurate the inference of departure time is. It could be taken for granted: the closer the capture time point was to the stable state, the less prior knowledge could be used for modeling, the worse the performance of the model would be. In this study, we think that it is unacceptable for the accuracy of capture time point to be lower than 15%, so that the abnormal point can be regarded as a failure point determining the practicability of the model. That is to say, after the time of failure point, the accuracy of estimating the departure time is below 15%, which is unacceptable. Based on the principle of thermal balance, it is doubtless that there must be a correlation between failure point and ambient temperature. Therefore, in different ambient environments, there are different failure points. Failure points cannot be the same number in different thermal curves, which means the values of failure points are hard to confirmed.

In practice, it would be very difficult to inversely estimate the departure time of the standing people if the obtained temperature was after the failure point, which was caused by a too long time window. Therefore, we should take action to reduce adverse effects of this long time window. We advise that witnesses or victims should keep as calm as possible when they see stealing or other crimes happening in houses. The best time for police to collect thermalfoot print information is as soon as possible, therefore witnesses should immediately call the police. In addition, it is recommended that they should keep the scene intact so as to ensure the integrity of the thermalfoot print information remaining on the ground. When the police arrive, witnesses or victims should briefly state the effective information that can be used by the police and cooperate with the police to collect the thermalfoot print information, which can help solve the case. The possibility of inversely estimating the departure time range of perpetrators lies in obtaining the thermalfoot prints left on the ground at the crime scene by the police within 15 min. With the development of technology, we hope that instead of being limited to deduce time points by detecting the remaining thermal traces on the ground, thermalfoot print technology can inspire more novel ideas using similar principle to acquire more effective information, and accordingly build a safer social environment.

### 5.5. Influence of Standing Time

In actual applications, the standing time of perpetrators was uncertain. The results of our preliminary experiments demonstrated that the standing time has little influence on the trend of thermal curves: the trends of thermal curves are basically the same for the standing time of 30 s and 3 min. Consequently, although some subjects just stood for a short time, the thermalfoot curve left on the floor can still provide us useful information for modeling, thus estimating the departure time.

## 6. Conclusions

In conclusion, the thermalfoot models could be developed based on heat traces can be used to estimate the departure time. According to Newton’s Law of Cooling, the thermalfoot models are able to be developed using the likelihood function, and correlation coefficients of these models are basically above 0.99 indicating that our algorithm has an excellent goodness of fit. In validation experiments, the residuals between estimated departure time points and ground-truth times are almost within a range of −500 s to 500 s. To further evaluate the performance of the thermalfoot model for the departure time reversal in actual application, the model’s reverse accuracy is calculated to determine whether the reverse time is within the acceptable time interval. The reverse accuracy of the thermalfoot model for estimating departure time at one-third, one-half, two-thirds, three-fourths, four-fifths, and five-sixths capture time points are 71.96%, 50.47%, 42.06%, 31.78%, 21.70%, and 11.21%, respectively. The results of comparison experiments with two subjective evaluation methods further demonstrate the effectiveness of thermalfoot model for the departure time reversal. In a short time window after someone leaves, the thermalfoot model has an excellent performance on the departure time reversal, whereas in a long time window after someone leaves, it is probably only about 15% to accurately determine the departure time using thermalfoot model. The thermalfoot model can help the police to solve crimes to some extent, which in turn brings more guarantees for people’s healthy life and social security and stability. The future work can focus on the influence of outliers, ROI selection, ROI size and different capture time points on the performance of thermalfoot model on departure time reversal. Also, we can explore the performance of thermalfoot model under the conditions where the environment temperature is below 16 ${}^{\circ }$C.

## Figures and Tables

**Table 1:** Comparison with two subjective calculation methods. (Subjective calculation${}^{1}$ and subjective calculation${}^{2}$ indicate the experts do not use and use auxiliary means such as ruler, respectively.)

Algorithm or Method	One-Third	One-Half	Two-Thirds	Three-Fourths	Four-Fifths	Five-Sixths
Thermalfoot model	71.96%	50.47%	42.06%	31.78%	21.70%	11.21%
Subjective calculation${}^{1}$	58.89%	37.38%	34.58%	23.36%	23.36%	11.21%
Subjective calculation${}^{2}$	62.62%	40.19%	36.45%	32.71%	28.04%	12.15%

**Table 2:** Outlier ratio of two situations: known starting point and predicted starting point under ambient temperatures of 16 °C and 28 °C.

Situation	16 °C	28 °C
Known starting point	14.29%	13.85%
Predicted starting point	26.29%	29.23%
